# Detection of Furrundu phlebovirus in *Aedes scapularis* (Diptera: *Culicidae*) collected in urban parks, in a highly urbanized city

**DOI:** 10.1590/S1678-9946202567038

**Published:** 2025-06-27

**Authors:** Gabriel Matheus do Nascimento, Camila Malta Romano, Andrei Rozanski, Thaís de Moura Coletti, Paulo Roberto Urbinatti, Delsio Natal, Mauro Toledo Marrelli, Alessandra Bergamo de Araújo, Marcello Schiavo Nardi, Antonio Charlys da Costa, Lícia Natal Fernandes

**Affiliations:** 1 Universidade de São Paulo, Faculdade de Medicina, Instituto de Medicina Tropical de São Paulo, Laboratório de Investigação Médica (LIM-49), São Paulo, São Paulo, Brazil; 2 Universidade de São Paulo, Faculdade de Medicina, Hospital das Clínicas, São Paulo, São Paulo, Brazil; 3 Universidade de São Paulo, Faculdade de Medicina, Instituto de Medicina Tropical de São Paulo, São Paulo, São Paulo, Brazil; 4 Universidade de São Paulo, Faculdade de Saúde Pública, Departamento de Epidemiologia, São Paulo, São Paulo, Brazil; 5 Prefeitura de São Paulo, Centro de Controle de Zoonoses, Laboratório de Fauna Sinantrópica, São Paulo, São Paulo, Brazil; 6 Secretaria do Verde e Meio Ambiente, Divisão da Fauna Silvestre, Coordenadoria de Gestão de Parques e Biodiversidade, São Paulo, São Paulo, Brazil

**Keywords:** Mosquito, Insect-specific virus, Phenuiviridae, High-throughput sequencing

## Abstract

Mosquitoes (Diptera: *Culicidae*) are arthropods of medical importance because they can carry arboviruses. High-throughput sequencing (HTS) technology and metagenomic approaches conducted in mosquitoes have contributed to the discovery of many insect-specific viruses (ISVs), which have the potential to affect their vector competence. Mosquitoes were collected in urban parks in Sao Paulo city, Brazil and 20 pools with female mosquitoes were subjected to HTS by HiSeq 2500 sequencing system (Illumina). Long viral sequences (1,585-6,701 base pairs) were recovered from two pools of *Aedes scapularis*. BLASTx analyses revealed they had greater identity with segment L and S of Salarivirus and segment M of Furrundu phlebovirus, which encode, respectively, the RNA-dependent RNA polymerase (RdRd), the nucleocapsid protein, and a polyprotein. Phylogenetic tree of the segment L and S of the *Phenuiviridae* Family showed our sequences grouped with unverified sequences of Furrundu phlebovirus, an unclassified ISV that belongs to the *Hareavirales* Order and was first reported in mosquitoes in the Brazilian *Pantanal*, the largest natural tropical wetland worldwide. We report the second detection of Furrundu phlebovirus in mosquitoes collected in urban parks, showing it could be in mosquitoes from natural places and in green areas in urban cities. We conclude that Furrundu phlebovirus possibly occurs in *Aedes scapularis* in green areas, in Sao Paulo. Further studies should elucidate the role of this virus in the vector competence of *Aedes scapularis* and its interaction with different arboviruses.

## INTRODUCTION

Mosquitoes (Diptera: *Culicidae*) are arthropods of medical importance because they can be vectors of arboviruses (arthropod born viruses). Such viruses cause diseases in humans and numerous outbreaks worldwide, such as yellow fever virus (YFV), dengue virus (DENV), Zika virus (ZIKV), Chikungunya virus (CHIKV), Japanese encephalitis virus (JEV), Venezuelan equine encephalitis virus (VEEV), West Nile virus (WNV), and others^1^.

Insect-specific viruses (ISVs) are also commonly encountered in mosquitoes. ISVs do not replicate in mammalian cells and can only replicate in insect cell lines^2^.

Recently, high throughput sequencing technologies enabled the discovery of many new ISVs in mosquitoes, enabling the use of metagenomic approaches, and enhancing knowledge about mosquito microbiome^3-5^. Some of the ISVs are phylogenetically related to arboviruses and have been classified in the families: *Flaviviridae, Togaviridae*, *Peribunyaviridae, Phenuiviridae*, *Rhabdoviridae*, and *Reoviridae*. Additionally, other ISVs, which are not phylogenetic related to arboviruses, have also been discovered and classified in the families: *Mesoniviridae, Tymoviridae*, *Birnaviridae, Totiviridae*, *Partitiviridae, Iflaviridae*, *Chuviridae, Circoviridae*, and to the taxon Negevirus^6,7^.

Recently, ISVs have gained attention due to the possibility that many arboviruses evolved from ISVs and that existing ISVs could jump from insects and infect vertebrates and evolve into new arboviruses^7^. Furthermore, ISVs have potential of modulating vector competence of mosquitoes in arboviruses transmission. The explanation is the superinfection exclusion, a mechanism in which similar viruses infecting a mosquito compete for the same resources^8-11^. Therefore, an ISV infection could affect the capacity of mosquitoes to be infected by arboviruses and to hold and transmit them, affecting vector competence^6,7^.

Some studies have investigated the effect of ISVs on the infection, dissemination, and/or transmission of pathogenic arboviruses in mosquitoes. Hall-Mendelin *et al*.^11^ intrathoracically inoculated an ISV, Palm Creek virus (PCV), in *Culex annulirostris* and later exposed them to a blood meal containing West Nile virus (WNV). The infected sample was infected with WNV and transmission rates were significantly lower in PCV-infected mosquitoes compared to non-infected ones . Romo *et al*.^12^ reported that *Aedes aegypti* inoculated with Nhumirim virus (NHUV), an ISV, showed lower ZIKV infection rates than that observed in mosquitoes not infected with NHUV. Furthermore, Baidaliuk *et al*.^13^ demonstrated that a prior infection by cell-fusing agent virus (CFAV) reduced the dissemination titer of ZIKV and DENV in mosquito head tissues.

Also, susceptibility of field mosquitoes to a certain arbovirus could be enhanced by previous ISV infections, as described by Olmo *et al*.^14^. The authors analyzed the virome of 515 specimens of *Aedes aegypti* collected in a dengue endemic area and observed that DENV infection in mosquitoes were more frequent when they were also infected by two other viruses, Phasi Charoen-like virus (PCLV) and Humaita-Tubiacanga virus (HTV). Laboratory experiments suggest that double infection of PCLV and HTV enhances the expression of histone H4, a pro-viral factor, that boosts DENV and ZIKV replication.

Therefore, knowledge on diversity and abundance of ISVs in mosquitoes from specific localities could help to predict arboviruses outbreaks and guide preventive actions^15^.

Detecting and sequencing ISVs in field-collected mosquitoes are important to raise information on their frequency, distribution, mosquito host range, genetic variability, evolution, and role in the vector competence of mosquitoes. Therefore, during a study to detect *Orthoflavivirus* in mosquitoes collected in parks in Sao Paulo city, some specimens were selected to investigate the viral diversity of ISVs by metagenomics. We report our main finding, the detection of Furrundu phlebovirus in *Aedes scapularis*.

## MATERIAL AND METHODS

### Study area

**Figure 1:**
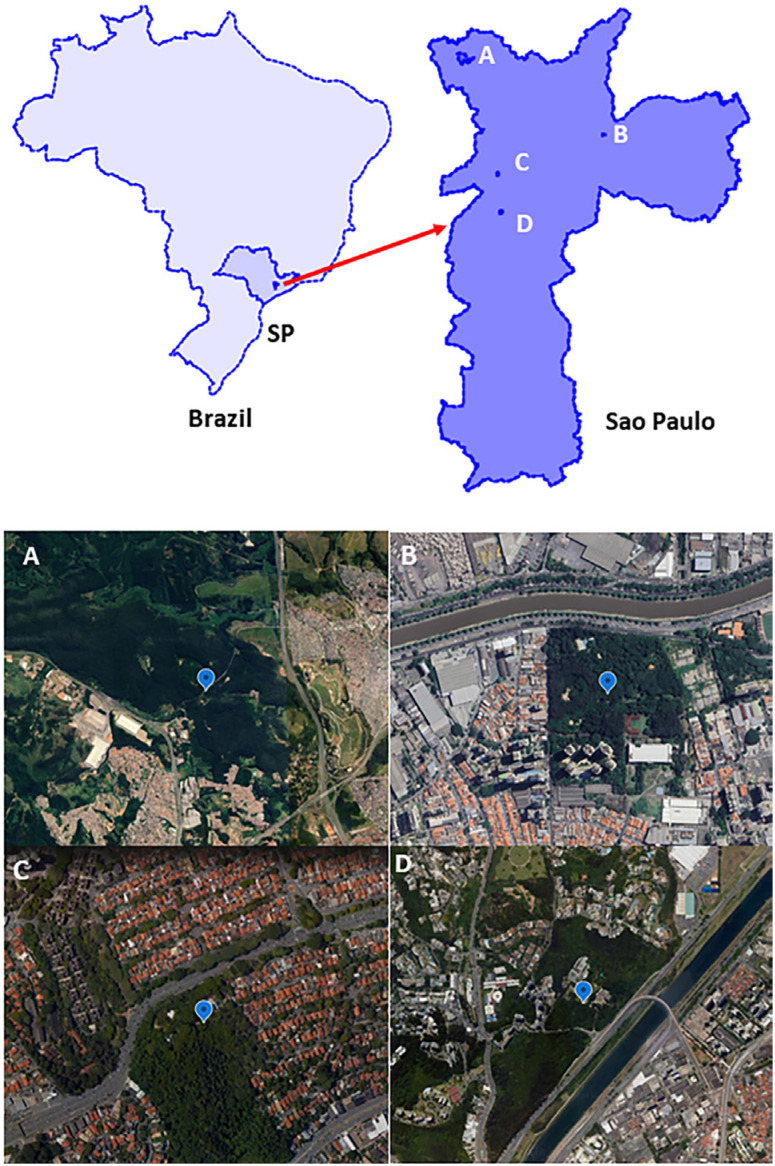
Study area. In the top: Map of Brazil showing the location of the Sao Paulo State (SP), with a dot representing the city of Sao Paulo. The red arrow points to the map of the city of Sao Paulo showing the location of the parks where mosquitoes were collected. In the bottom: Parks and their surrounding areas. A = Anhanguera, B = Piqueri, C = Previdencia, D = Burle Marx.

Mosquitoes (Diptera: *Culicidae*) were collected in urban parks in the Sao Paulo city, Sao Paulo State, located in the Southeast of Brazil (Figure 1). The city has over 12.3 million people and is a highly urbanized area, in which parks are among the last places for biodiversity protection and conservation^16^.

Four parks were selected for this study: Anhanguera, Burle Marx, Piqueri, and Previdencia. Their areas and geographical coordinates are, respectively: 9,500,000 m2/23º25’130"S, 46º46’958"W; 480,000 m2/23°37’56"S 46°43’17"W; 98,000 m2/23°31’39.98"S 46°34’24.88"W, 44,323 m2/23°34’50"S 46°43’36"W. The vegetation consists mainly of remnants of the native Atlantic Forest, with artificially planted trees (mainly eucalypts), gardens, and grassy areas. A great diversity of birds is most prominent among several species of animals inhabiting these parks, which are widely used for sports and leisure activities^17^.

### Collection and identification of mosquitoes

During an entomo-virological investigation, mosquitoes were collected once a month in each park from August 2012 to July 2013. The collection were performed using a Nasci aspirator, CDC-CO_2_ and Shannon trap, as described elsewhere^18^. The mosquitoes were transported to the laboratory in dry ice and stored at −80 °C until they were used. Morphological identification was performed at approximately −10 °C on a specially designed chilled table using a stereo microscope and the dichotomous keys of Forattini^19^ and Consoli and Oliveira^20^. The mosquitoes were identified to the specie level, or, at least, to genus level. Up to 10 non-engorged females were grouped in pools according to their taxon, place, and date of collection. The mosquitoes were stored in a −80 °C freezer after identification. Twenty pools were selected for this study.

### Nucleic acids extraction

Pools with non-engorged female mosquito were thawed and homogenized in 1 mL Gibco^®^ Hanks’ buffered salt solution (HBSS) with a Pellet Pestle Motor (Sigma-Aldrich). Micro tubes (1.5 mL) containing the samples were centrifuged (12,000 x g/2 min/4 °C) and the supernatant (400 μL) was subjected to the Specific B/Lysis off board protocol of NucliSENS^®^ easyMag^®^ (BioMérieux), according to the manufacturer's instructions. Nucleic acids were eluted in 80 µL of elution buffer and stored at −80 °C.

### Sample processing and high-throughput sequencing (HTS)

HTS was performed according to Costa *et al*.^21^. In summary, each mosquito pool was added to a tube containing lysing matrix C (MP Biomedicals, USA) and homogenized in 900 μL of HBSS in a FastPrep-24 5G Homogenizer (MP biomedicals, USA). The homogenized sample was centrifuged at 12,000 × g for 10 min, and 300 μL of the supernatant was filtrated through a 0.45 μM filter (Merck Millipore, Billerica, MA, USA). A total of 100 μL of cold PEG-it Virus Precipitation Solution (System Biosciences, CA, USA) was added to the filtrate, and the samples were gently mixed and incubated at 4 °C for 24 h. The samples were centrifuged at 10,000 × g for 30 min at 4 °C, the supernatant (∼350 μL) was discarded and the pellet was treated with nuclease enzymes (TURBO DNase and RNase Cocktail Enzyme Mix-Thermo Fischer Scientific, CA, USA; Baseline-ZERO DNase - Epicentre, WI, USA; Benzonase-Darmstadt, Germany; and RQ1 RNaseFree DNase and RNase A Solution-Promega, WI, USA). The resulting mixture was incubated at 37 °C for 2 h. Viral nucleic acids were extracted using ZR & ZR-96 Viral DNA/RNA Kit (Zymo Research, CA, USA) according to the manufacturer's instructions. The cDNA was synthesized with AMV Reverse transcription (Promega, WI, USA) and converted to double strand of cDNA using DNA Polymerase I Large (Klenow) Fragment (Promega, WI, USA). Subsequently, a Nextera XT Sample Preparation Kit (Illumina, CA, USA) was used to construct a DNA library, which was identified using dual barcodes. Individual samples were purified using ProNex Size-Selective Purification System (Promega, WI, USA). For size range, Pippin Prep (Sage Science, Inc.) was used to select a 300 bp insert (range 200–400 bp). The library was deep sequenced using the HiSeq 2500 Sequencer (Illumina, CA, USA) with 126 bp ends. Bioinformatic analysis was performed according to Deng *et al*.^22^. The resulting singlets and contigs were analyzed using BLASTx to search for similarity to viral proteins in GenBank's Virus RefSeq. The contigs were compared to the GenBank non-redundant nucleotide and protein database (BLASTn and BLASTx). All the nucleotide sequences were deposited in the GenBank under the following accession numbers (PV246954 to PV246961). Conserved motifs were detected from amino acid (aa) sequences alignment performed with Bio Edit.

### Phylogenetic analyses

Multiple sequence alignment was performed with Bio Edit using sequences generated in this study and sequences retrieved from GenBank. A maximum likelihood tree was reconstructed using IQ-TREE (version 1.6.12, Center for Integrative Bioinformatics Vienna, University of Vienna, Vienna, Austria) under the GTR+R+R8 nucleotide substitution model chosen as the best fitting model according to the Bayesian information criteria via Model Finder. Bootstrap support was done with 1,000 replicates. Phylogenetic trees were visualized by Figtree (version 1.4.4). Genetic distances were estimated by MEGA (version 11, Institute for Genomics and Evolutionary Medicine, Temple University, Philadelphia, Pennsylvania, USA).

## RESULTS

Twenty mosquito pools (150 mosquitoes) from different taxa and localities were analyzed by HTS. Supplementary Table 1 shows information about taxon of mosquitoes, the number of specimens in each pool, place, and date of collection of the analyzed pools.

The analysis of the consensus sequences obtained by HTS revealed the detection of short viral contigs in most mosquito pools (data not published), except for two pools of *Aedes scapularis* (samples 4 and 13), in which long contigs, with 1,000 base pairs (bp) or more, were detected. BLASTx analyses revealed that our long sequences had greater identity with one of the following sequences of unclassified viruses of the order *Hareavirales*: Salarivirus Mos8CM0 (API61884.1 and API61886.1), Salari virus (QGA70945.1) or Furrundu phlebovirus (QFS19689.1). Short sequences showing greater identity with Salarivirus Mos8CM0 (API61884.1) were detected in samples 4, 13, and 14. Table 1 shows the information about the mosquito pools and the nucleotide sequences detected in each pool. Supplementary Figure S1 shows BLAST alignments of our aa sequences and the GenBank sequences of the different segments of the Salari virus Mos8CM0 isolate.

**Table 1:** Pools of *Aedes scapularis* in which partial nucleotide sequences of viral segments were detected.

Sample number	Collection date	Number of mosquitoes/pool	Collection place (Park)	Nucleotide sequences detected	Blastx best hit
				Size in bp (GenBank accession number)	(Encoded protein/viral segment)
4	04/03/2013	6	Anhanguera	1923 (PV364151)	Salarivirus Mos8CM0 (Nucleocapside/S)
				6701 (PV246954)	Salarivirus Mos8CM0 (RdRp/L)
13	25/03/2013	10	Previdencia	1585 (PV246961)	Furrundu phlebovirus (polyprotein/M)
				2615 (PV246955)	Salari virus (RdRp/L)
				2064 (PV246956)	Salarivirus Mos8CM0 (RdRp/L)
				717 (PV246957)	Salarivirus Mos8CM0 (RdRp/L)
14	08/04/2013	10	Burle Marx	344 (PV246958)	Salarivirus Mos8CM0 (RdRp/L)
				261 (PV246959)	Salarivirus Mos8CM0 (RdRp/L)

bp = base pair; RdRp = RNA-dependent RNA polymerase

**Figure 2:**
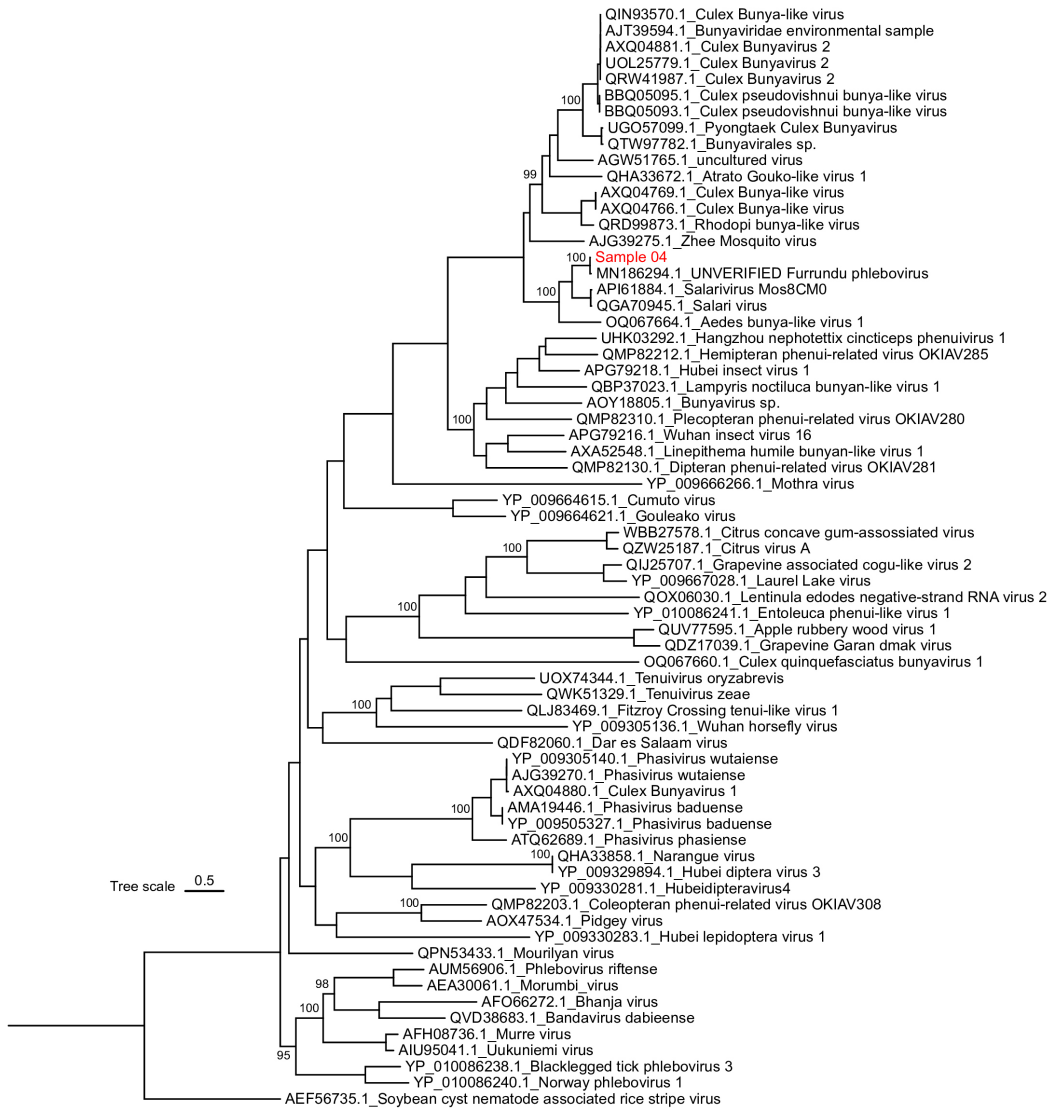
Maximum likelihood phylogenetic tree of a partial Segment L (1,732 aa) of *Phenuiviridae* Family. The sequence generated in this study is red (see additional information in Table 1). The sequences retrieved from GenBank are black (represented with their GenBank accession numbers and virus name or taxonomic classification).

**Figure 3:**
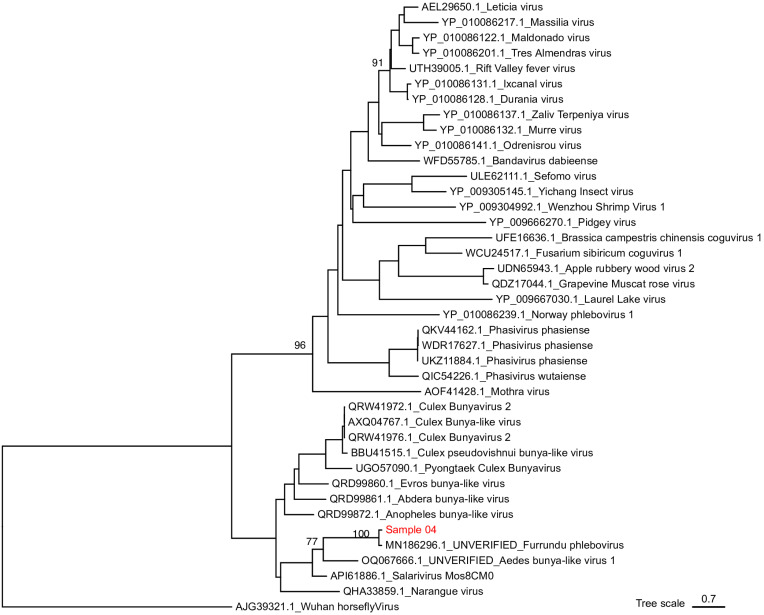
Maximum likelihood phylogenetic tree of a partial Segment S (291aa) of *Phenuiviridae* Family. The sequence generated in this study is red (see additional information in Table 1). The sequences retrieved from GenBank are black (represented with their GenBank accession numbers and virus name or taxonomic classification).

The longest RdRp nucleotide sequence and the only nucleocapsid sequence (sample 4) were respectively chosen to be used in phylogenetic analyses of segment L and S of the virus. Phylogenetic tree of the segment L (partial) of the family *Phenuiviridae* (order *Hareavirales*) was reconstructed with the consensus sequence of sample 4 and other 67 viral sequences retrieved from GenBank. Our sequence, which had higher identity with Salarivirus according to BLASTx analyses, grouped with an unverified sequence of a virus named Furrundu phlebovirus (Figure 2). Phylogenetic analyses of the segment S (partial) of the *Phenuiviridae* Family, which were conducted with our consensus sequence and other 39 sequences retrieved from GenBank, also showed that our sequence had closer relationship with an unverified sequence of Furrundu phlebovirus (Figure 3). Good statistical support was observed in both cases, with bootstrap values of 1,000.

The aa distance matrix obtained by Mega showed our sequences shared similarity of 98,4% and 88,9% with segments L and S, respectively, of the Furrundu phlebovirus sequence from GenBank, whereas the similarity shared with sequences of the respective segments of Salarivirus was 70% and 36%.

**Figure 4:**
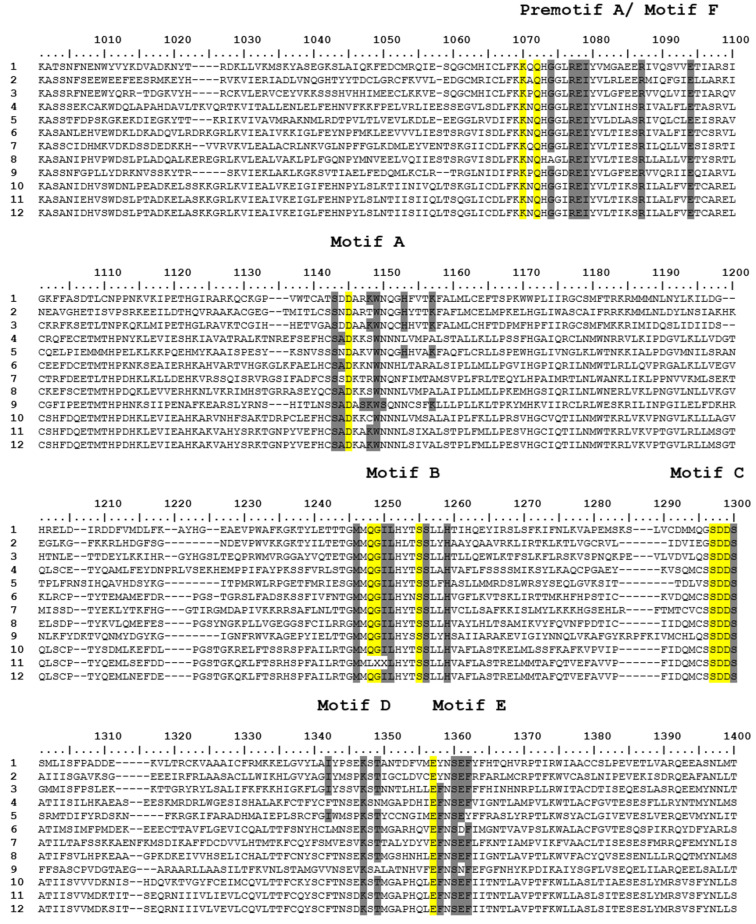
Alignment of a partial aa sequence of the segment L of *Hareavirales*, showing conserved motifs of the RdRp. Conserved motifs are highlighted in yellow and less conserved motifs are in gray. Numbers in the left side of the alignment refer to GenBank sequences of arboviruses (1-3), classified and unclassified insect-specific viruses (4-5 and 6-11, respectively), and the sequence detected in this study (12). 1:AUM56906.1/Rift Valley fever phlebovirus, 2: AFO66272.1/ Bhanja virus, 3: AFH08736.1/ Murre virus, 4: QHA33858.1/ Narangue virus, 5: YP009664621.1/ Goleako virus, 6: AJG39275.1/ Zhee Mosquito virus, 7: APG79218.1/ Hubei insect virus1, 8: UGO57099.1/ Pyongtaek Culex Bunyavirus, 9: AJG39270.1/ Wutai mosquito phasivirus, 10: API61884.1/ Salarivirus Mos8CM0, 11: MN186294.1/ UNVERIFIED: Furrundu phlebovirus, 12: XPT04934.1/ Sample 04.

The alignment of partial aa sequences, performed with GenBank sequences of *Hareavirales* and the sequence from sample 4, shows the presence of the conserved motifs of the RdRp coding region: pre-motif A/motif F and motifs A, B, C, D, and E (Figure 4).

## DISCUSSION

We detected nucleotide sequences of Furrundu phlebovirus in *Aedes scapularis* collected in parks, in Sao Paulo city. Our findings indicate this virus might occur in *Aedes scapularis* in urban parks, in Sao Paulo.

To our knowledge, this is the second report about the detection of this virus worldwide. Furrundu phlebovirus was first reported in Brazil^23^. The authors performed metagenomics in salivary glands of mosquitoes collected in the North of the Brazilian Pantanal and detected partial sequences in two pools of *Aedes scapularis*. The sequences shared similarity of 40, 30, and 62%, respectively, with the segments S, M, and L of Salarivirus Mos8CM0. The authors suggested the sequences belonged to a putative novel ISVs named Furrundu phlebovirus, *Phenuiviridae* Family.

In our phylogenetic studies, Furrundu phlebovirus is assembled with many unclassified viruses related to the *Phenuiviridae* Family. Our distance matrix, which performed with all the sequences used to reconstruct the phylogenetic tree of segment L, shows Furrundu phlebovirus shares similarity of 28 to 44% with unclassified viruses. Additionally, among the classified viruses, Furrundu phlebovirus shared the highest similarity values (22-23%) with *Bandavirus, Mobuvirus*, *Uukuvirus, Wenrivirus,* and *Goukovirus*. Therefore, in the light of current data, we understand it is not possible to suggest the inclusion of Furrundu phlebovirus into any of the described genus in the *Phenuyviridae* Family. Once new viral sequences become available, future phylogenetic studies will be able to answer this issue. However, the presence of conserved motifs of the RdRp coding region in our sequence, as well as in *Phenuyviridae* sequences (Figure 4) reinforces that Furrundu phlebovirus is related to this Family.

According to the International Committee on Taxonomy of Viruses^24^, the *Phenuiviridae* Family belongs to the Order *Hareavirales*, formerly *Bunyavirales*, and currently shows 23 genera and 159 viral species. The segmented genome consists of two to eight segments of negative sense or ambisense single strand RNA, with the total size ranging from 8,1 to 25,1kb. The number of proteins encoded in this Family vary among different genus and species. Most viruses of this Family encode structural proteins: the RdRp, which acts in replication and transcription of viral genomic RNA and is encoded by segment L (6,3 to 9,5 Kb); the precursor polypeptide, which originates the two envelope glycoproteins (Gn and GC) and are encoded by segment M (1,6 to 5,1kb); and the nucleocapsid protein N, which is encoded by segment S (1 to 2,7kb). The non-structural proteins NSm and NSs are also encoded by segments M and S, respectively, of some phenuivirids. In addition, other non-structural proteins are encoded by different segments of some *Phenuiviridae*.

*Phenuiviridae* is a large Family, with new members frequently discovered and great diversity among the comprised viruses. Phenuivirids can infect vertebrates (humans, livestock, birds), crustaceans, arthropods (mosquitoes, sandflies, ticks), plants, and fungi. Many of them are arboviruses and replicate in vertebrates and arthropods. They can be pathogenic, causing burdens to human health, livestock, and agriculture, such as *Dabie bandavirus*, a tick-borne virus that causes the severe fever thrombocytopenia syndrome (SFTF) in humans and animals, *Rift Valley fever phlebovirus*, a mosquito-borne zoonosis that affects humans and animals and *Rice stripe tenuivirus*, which reduces rice yelds in up to 40%^25,26^.

Furrundu phlebovirus was detected in *Aedes scapularis*, the same mosquito specie in which this virus was previously detected^23^. *Aedes scapularis* is distributed in the Americas, especially in South America^19^. This species occurs in natural environments and in rural and urban places^27,28^. It was considered a suspect vector of Rocio virus (ROCV), responsible for an epidemic in some municipalities of the southeast region of Brazil, in the 1970's^29^. *Aedes scapularis* has been found naturally infected by YFV^30-32^. This specie is also a competent vector of Venezuelan Equine Encephalitis Virus (VEEV)^33^. In addition to medically important arboviruses, an ISV, Guapiacu virus (GUAPV), was isolated in *Aedes scapularis* collected in the city of Guapiacu, Sao Paulo State^34^, reinforcing the occurrence of ISVs in this species.

Once *Aedes scapularis* is a competent vector of some arboviruses and a potential vector of other arboviruses, the knowledge on the ISVs that occur in this host is important. According to some studies, a previous ISVs infection has the potential to difficult or inhibit a second infection by a medically important arbovirus in mosquitoes, affecting their vector competence, as reported^11-13^. We report the occurrence of Furrundu phebovirus, an ISVs, in *Aedes scapularis* collected in Sao Paulo city. We highlight the importance of studying this virus to obtain data on its frequency, distribution, mosquito host range, and role in the vector competence of *Aedes scapularis*.

In Sao Paulo city, *Ae. scapularis* has been detected in different localities^35-38^. So far, there is one report of YFV detected by Reverse Transcription-Polymerase Chain Reaction (RT-PCR) in *Aedes scapularis* collected in this city^32^. To our knowledge, no other arbovirus or ISVs has been detected in *Aedes scapularis* in Sao Paulo.

Here we detected Furrundu phlebovirus in *Aedes scapularis* collected in three different urban parks: Anhanguera, Previdencia, and Burle Marx, which are in the Northwest, West, and South regions of the city, respectively. Anhanguera Park is 33 km from Burle Marx Park and 26 km from Previdencia Park. Previdencia and Burle Marx Parks are 10 km apart. Therefore, our findings suggest it is possible that Furrundu phlebovirus is distributed in different regions of Sao Paulo city.

Maia *et al*.^23^ have detected Furrundu phlebovirus in Pantanal, the largest natural tropical wetland, a very diverse biome with a great variety of vertebrates and invertebrates. We detected Furrundu phlebovirus in urban parks, showing the virus could be in mosquitoes from natural places and in green islands among urban densification, like the urban parks in the Sao Paulo city.

## CONCLUSION

We conclude that Furrundu phlebovirus, an unclassified virus that belongs to the *Hareavirales* Order and *Phenuiviridae* Family, might occur in *Aedes scapularis* in green areas, in Sao Paulo city. Further studies should raise data on the frequency and distribution of this virus in field collected mosquitoes and elucidate the role of this virus in the vector competence of *Aedes scapularis*.

## Appendix

Supplementary Material available from: https://doi.org/10.48331/scielodata.V9WPVP
