# Supplementary material for: Detection of Furrundu phlebovirus in Aedes scapularis (Diptera: Culicidae) collected in urban parks, in a highly urbanized city
Source: Rev Inst Med Trop Sao Paulo. 2025 Jun 27;67:e38. doi: 10.1590/S1678-9946202567038 (PMC12204209; doi:10.1590/S1678-9946202567038)
Supplement: Supplementary file 1 [file 1678-9946-rimtsp-67-S1678-9946202567038-suppl.pdf]

REVISTA DO INSTITUTO DE MEDICINA TROPICAL DE SÃO PAULO

JOURNAL OF THE SÃO PAULO INSTITUTE OF TROPICAL MEDICINE

SUPPLEMENTARY MATERIAL

https://doi.org/10.48331/scielodata.V9WPVP

Detection of Furrundu phlebovirus in *Aedes scapularis* (Diptera: *Culicidae*) collected in urban parks, in a highly urbanized city

Gabriel Matheus do Nascimento[1], Camila Malta Romano[2], Andrei Rozanski[3], Thaís de Moura Coletti[1], Paulo Roberto Urbinatti[4], Delsio Natal[4], Mauro Toledo Marrelli[4], Alessandra Bergamo de Araújo[5], Marcello Schiavo Nardi[6], Antonio Charlys da Costa[3*], Lícia Natal Fernandes[1*]

[1]Universidade de São Paulo, Faculdade de Medicina, Instituto de Medicina Tropical de São Paulo, Laboratório de Investigação Médica (LIM-49), São Paulo, São Paulo, Brazil

[2]Universidade de São Paulo, Faculdade de Medicina, Hospital das Clínicas, São Paulo, São Paulo, Brazil

[3]Universidade de São Paulo, Faculdade de Medicina, Instituto de Medicina Tropical de São Paulo, São Paulo, São Paulo, Brazil

[4]Universidade de São Paulo, Faculdade de Saúde Pública, Departamento de Epidemiologia, São Paulo, São Paulo, Brazil

[5]Prefeitura de São Paulo, Centro de Controle de Zoonoses, Laboratório de Fauna Sinantrópica, São Paulo, São Paulo, Brazil

[6]Secretaria do Verde e Meio Ambiente, Divisão da Fauna Silvestre, Coordenadoria de Gestão de Parques e Biodiversidade, São Paulo, São Paulo, Brazil

*These authors jointly supervised this study

Correspondence to: Lícia Natal Fernandes Universidade de São Paulo, Faculdade de Medicina, Instituto de Medicina Tropical de São Paulo, Laboratório de Investigação Médica (LIM-49), Av. Dr. Enéas Carvalho de Aguiar, 470, CEP 05403-000, São Paulo, SP, Brazil

E-mail: licianatal@usp.br

Received: 11 October 2024

Accepted: 28 April 2025

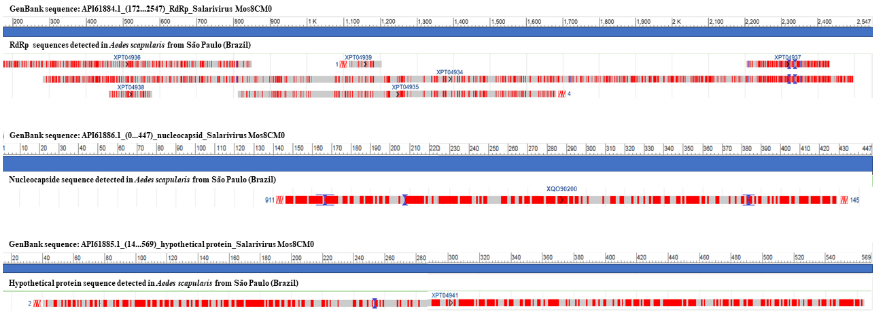

Supplementary Figure S1 - Graphic obtained by Blastx showing the alignment of the partial aa sequences of Furrundu phlebovirus detected in Aedes scapularis collected in São Paulo with GenBank sequences of the Salari virus Mos8CM0 isolate. Sequences from GenBank are represented in blue bars and information about them (accession number, size in aa, encoded protein and name of the viral isolate) is available above each bar. Red and gray bars represent the sequences of Furrundo phlebovirus detected in mosquitoes collected in Sao Paulo. Information about them (encoded protein and accession number) is available above the bars.

Supplementary Table S1 - Information about mosquito pools analysed by HTS according to the taxon of mosquitoes, number of specimens in each pool and collection place and collection date.

Sample (pool)	Taxonomic category	Number of mosquitos	Park	Date of collection
1	*Aedes aegypti*	1	Piqueri	21/02/2013
2	*Aedes albopictus*	4	Previdencia	22/04/2013
3	*Aedes fluviatilis*	10	Piqueri	18/03/2013
4	*Aedes scapularis*	6	Anhanguera	04/03/2013
5	*Aedes scapularis*	10	Piqueri	21/02/2013
6	*Anopheles fluminenses*	4	Anhanguera	03/12/2012
7	*Anopheles strodei*	10	Anhanguera	03/12/2012
8	*Coquillettidia sp.*	4	Anhanguera	01/04/2013
9	*Hemagogus leucocelaenus*	1	Anhanguera	04/02/2013
10	*Limatus durhami*	5	Anhanguera	04/02/2013
11	*Psorofora ferox*	10	Anhanguera	03/12/2012
12	*Uranotaenia pucherrima*	10	Anhanguera	01/10/2012
13	*Aedes scapularis*	10	Previdencia	25/03/2013
14	*Aedes scapularis*	10	Burle Marx	08/04/2013
15	*Anopheles strodei*	10	Burle Marx	08/04/2013
16	*Culex nigripalpus*	10	Piqueri	21/02/2013
17	*Culex nigripalpus*	10	Previdencia	25/03/2013
18	*Culex nigripalpus*	10	Anhanguera	04/03/2013
19	*Culex nigripalpus*	10	Burle Marx	11/03/2013
20	*Culex (Melanoconion)* seção Melanoconion	5	Anhanguera	01/10/2012

This is an open-access article distributed under the terms of the Creative Commons Attribution License.
